# Controversies in the Management of the Airway in Panfacial Fractures: A Literature Review and Algorithm Proposal

**DOI:** 10.3390/jcm13237294

**Published:** 2024-11-30

**Authors:** Antonio Marí-Roig, Niall M. H. McLeod, Jan De Lange, Leander Dubois, Maria Fe García Reija, Bauke Van Minnen, Harald Essig

**Affiliations:** 1 Department Oral and Maxillofacial Surgery, Hospital Universitari Bellvitge, 08907 Barcelona, Spain; 2 Department Oral and Maxillofacial Surgery, University Hospitals Coventry and Warwickshire, Coventry CV2 2DX, UK; niall.mcleod@nhs.net; 3 Department Oral and Maxillofacial Surgery, Amsterdam UMC, 1012 WX Amsterdam, The Netherlands; j.delange@amc.uva.nl (J.D.L.); l.dubois@amc.uva.nl (L.D.); 4 Department Oral and Maxillofacial Surgery, Hospital Universitario Marqués de Valdecilla, 39008 Santander, Spain; mf1971@hotmail.com; 5 Department Oral and Maxillofacial Surgery, University Medical Center Groningen, 9713 GZ Groningen, The Netherlands; b.van.minnen@umcg.nl; 6 Department Oral and Maxillofacial Surgery, University Hospital Zürich, 8032 Zürich, Switzerland; harald.essig@usz.ch

**Keywords:** airway management, panfacial fractures

## Abstract

Panfacial fractures are complex fractures involving multiple regions of the facial skeleton and may require multiple surgeries over a relatively short period. They are often associated with polytrauma and other injuries including neurotrauma, which require either immediate (ATLS) airway management, prolonged intubation, or repeated intubations for staged surgeries. The choice of airway for the surgical management of these fractures is difficult, as an assessment of the occlusion is required, and the central nasal complex and/or skull base may be involved, making classical orotracheal or nasotracheal intubation problematic. Submental intubation is increasingly reported as a method of airway management with the aim of avoiding a tracheostomy and its related complications. A review of the different techniques of airway management in the elective treatment of panfacial fractures was performed, focusing on the pros and cons of each method. Most articles were retrospective studies, with only one prospective study comparing submental intubation to tracheostomy in panfacial fractures. An algorithm for the management of the airway in panfacial fractures was presented, based on a sequential assessment of the existing airway, the surgical access required, and the need for prolonged or repeated intubation. Front of neck access, orotracheal and nasotracheal intubation, and submental intubation are all appropriate techniques in different circumstances, and the advantages and disadvantages of each are presented.

## 1. Introduction

Panfacial fractures are traditionally defined as those involving all three regions of the face: the frontal, midface, and mandible regions. The term, however, is commonly used for any facial fracture involving two or more of these areas. They are most often the result of high-energy trauma, and patients commonly have other injuries, most importantly intracranial and spinal injuries but also limb, thoracic, abdominal, and pelvic injuries [1,2].

The initial management of patients presenting with trauma now follows the well-established Advanced Trauma Life Support (ATLS) protocols, starting with the assessment and management of the Airway and Catastrophic Hemorrhage with the establishment of a definitive airway where appropriate [3].

In an emergency setting, this is normally achieved via orotracheal intubation (OTI) with cervical spine immobilization, with a high success rate when performed by trained rescue teams/anesthesiologists [4]. When this is deemed not feasible due to the severity of the facial/oral injury, the distortion of the anatomy, or severe hemorrhage, ‘front of neck access’ (FONA) to the trachea is the technique of choice. In these situations, cricothyroidotomy has been shown to be faster and have lower morbidity and mortality rates than tracheostomy. FONA is also the final step for “can’t intubate, can’t oxygenate” (CICO) emergencies, as stated in the Difficult Airway Society guidelines [5].

Orotracheal intubation is well suited for the acute management of the airway and for the intraoperative management of non-facial injuries, but in a dentate patient, it does not permit the establishment of the occlusion, one of the key outcomes sought in facial fracture treatment, hence the need to consider alternative options for the definitive surgery of panfacial fractures. Whether the patient is intubated already due to their injuries or not, the options for managing the airway intraoperatively include oral/nasal endotracheal intubation, submental intubation (SMI), or surgical FONA. The decision to perform one or another is based upon the patient’s fracture complexity and location; the type of surgery to be performed, including the need for maxillomandibular fixation and the involvement of the central nasal complex and skull base; and also the general status of the patient and the need for prolonged ventilation or planned phased surgeries [6].

In a survey on the airway management of maxillofacial trauma published in 1997 [7], tracheostomy was the first choice performed among surgeons and anesthesiologists for patients with panfacial fractures or those with loss of consciousness and midface fractures.

Trends have substantially changed since then, with the advent of new methods of intubation such as submental intubation [8,9]. In a retrospective study, Daniels et al. [10] reported that 86.1% of patients in a cohort of 43 panfacial fractures had SMI performed.

The objectives of this literature review on the management of the airway in panfacial fractures are to assess the different methods of airway management proposed, focusing on controversies, and secondarily to offer a potential algorithm for airway management.

## 2. Materials and Methods

A review of the literature pertaining to the management of the airway in maxillofacial trauma was undertaken in January 2023. A Search of the PubMed database for articles published until 31 December 2022 was performed with the following search terms: (*panfacial fracture*) *AND* (*airway management*). The search was limited to full-length articles on adult patients and English-language articles. Technical notes and case reports were excluded.

Following the full-text review, the areas of controversy and debate were identified.

## 3. Results

Article titles were screened for eligibility by AMR, and a total of 43 articles were identified and the abstracts collated. Abstracts were screened by AMR and NM for the inclusion criteria, and a total of 26 articles were identified for which the full text was obtained. (Excluded: single case report 7; Technical notes 4; Off topic 3; Letters to the editor 2; pediatric population 1). A manual search was conducted for the articles identified and their references, and another 32 additional relevant articles were extracted.

All articles were of low quality of evidence, being prospective or retrospective case series, with the vast majority focusing on SMI. Only one article [7] was a prospective, randomized, controlled study comparing SMI to tracheostomy

The key controversies determined in the selection of an adequate method of airway management in panfacial fractures include the usefulness of retromolar intubation [11]; the safety of nasotracheal intubation (NTI) in the context of a cranial base fracture [12]; the feasibility of the intraoperative switch from NTI to OTI in the context of a panfacial fracture [13]; the convenience of performing SMI in a comminuted anterior mandibular fracture [14]; the need for a tracheostomy if a patient will not need a prolonged intubation and also its reported intraoperative and postoperative complications [15]; the role of cricothyroidotomy in the elective management of panfacial fractures [16]; and lastly, the staged surgical management of panfacial fractures that may avoid the mentioned pitfalls in airway management.

## 4. Discussion

The options for securing a definitive airway when a patient with a panfacial fracture arrives at an operating room are multiple: oral/nasal endotracheal intubation (oral, oral followed by retromolar intubation, nasal, nasal followed by oral), SMI, or surgical FONA (surgical or percutaneous tracheostomy and cricothyroidotomy).

Patients may present to the operating theater with an orotracheal tube (OTT), a tracheostomy, or a cricothyroidotomy in situ from their primary trauma management, and a decision then still needs to be made on how to proceed.

### 4.1. Retromolar Intubation

In the management of facial fractures, an OTT would prevent the assessment of the occlusion, interfere with the reduction of maxillary and mandibular fractures, and preclude intermaxillary fixation (IMF).

If an OTT is already in situ, one option is to leave it in place and switch to retromolar intubation. This is a useful alternative that avoids the need to remove the existing airway, particularly when reintubation may be difficult [17]. The main advantage is that retromolar intubation is an easy technique to perform and takes very little time [17].

Retromolar intubation was first described by Bonfils in 1983 [18] as a new method for difficult intubation in a Pierre Robin case but was popularized by Martinez-Lage in 1998 primarily for orthognathic surgery and craniofacial surgeries [19]. His technique included the creation of space for the OTT in the retromolar area either with the removal of third molars when present and/or a semilunar osteotomy large enough for the tube to lie below the occlusal plane. Retromolar fiberoptic OTI for cases of severe trismus has also been described [20,21].

Attempts have been made to measure the available space in the retromolar area to ensure there is space for the OTT to sit. Sittitavornwong et al. [11] retrospectively reviewed the CT scans of maxillofacial trauma patients and compared the area to different reinforced endotracheal tubes and concluded that the retromolar space area was statistically significantly larger than the reinforced oral endotracheal tube area for sizes 6.0, 6.5, and 7.0. A limitation of this study was that it included only patients who were missing third molars or had their third molars impacted, which would not affect the available retromolar space, but also, the soft tissue in the retromolar area, particularly along the ascending ramus and tuberosity area, was not considered.

Retromolar intubation has disadvantages such as interference by the OTT within the surgical field and is not feasible where there is limited bony retromolar space or if there is an excess of soft tissue in the retromolar trigone. Retromolar intubation may not be suitable when mandibular angle fractures are present as it may interfere with the surgical approach, reduction, and/or osteosynthesis. Finally, a suitably flexible orotracheal tube must be used that can bend without kinking.

The complications reported for retromolar intubation are the displacement of the tube and interference in the surgical field [17].

### 4.2. Is Nasotracheal Intubation Contraindicated in the Presence of a Skull Base Fracture?

The most common technique for airway management in facial fractures where there is a need to assess the occlusion is the use of a nasal airway [22,23]. In a survey by Smoot et al. [7], more than 50% of surgeons and anesthesiologists chose some form of NTI for fracture patterns involving the midface.

Most of the concerns expressed by anesthesiologists for NTI were related to fracture patterns in which the cribriform plate status was unknown, or there were fractures of the ethmoids or basilar skull or fractures designated Le Fort II or III. These concerns were based on anecdotal reports of intracranial intubation and cerebrospinal fluid leakage following NTI [12]. Large retrospective case series, however, have demonstrated the safety of NTI even in the presence of a skull base fracture (SBF) [24,25] in both an acute setting and elective management. In a study of 86 patients with SBFs by Rhee et al. [26], there were no differences when comparing blind NTI to OTI in terms of CSF leakage, meningitis, cranial nerve injury, or intracranial placement. Jazayeri-Moghaddas et al. [27] analyzed three groups of patients depending on the method of intubation (NTI only, OTI only, and patients who were initially nasally intubated and changed to an oral tube after admission) and found no differences in mortality for the three groups. Both the NTI and OTI group had fewer cases of sinusitis and pneumonia than the combination group, and no patient had sinus or cribriform plate penetration regardless of the intubation method. In a study of 160 patients with an SBF and CSF fistula, Bahr and Stoll [24] reported that the route of tracheal intubation had no influence on the postoperative complication rate. There was no case of direct cerebral injury associated with NTI, and the incidence of meningitis was the same, 2.5%, after oral and nasal intubation. The authors concluded that NTI was not contraindicated in the presence of frontobasal fractures. Despite this, concerns about NTI in the presence of ACF fractures remain widespread.

Because fractures involving the central midface will distort the normal anatomy, Rosen et al. [28] recommend that although blind nasal intubation is safe, in a controlled environment such as the operating room, NTI should be performed under direct vision with fiberoptic guidance [17].

NTI can, however, also cause problems in the management of facial fractures involving the nasoethmoid complex beyond the additional difficulty for the insertion of an NTT. When the nasoethmoid complex needs to be repaired, this nasotracheal tube can jeopardize the correct reduction of fractures, and in these scenarios, an intraoperative change from an NTT to an OTT has been advocated for [13]. Moreover, NTI usually involves passing the tube and securing it over the top of the patient’s head, and so, if a coronal flap is indicated for access to the midface or frontal regions, this will get in the way. Although the tube can be placed across the midface, this still has the potential to interfere with the surgical procedure.

In summary, NTI is not contraindicated in the presence of a skull base fracture, even in acute management. To avoid the rare but devastating consequences of intracranial intubation, fiberoptic intubation is recommended.

### 4.3. Nasotracheal to Orotracheal

To overcome the situation of a patient with panfacial trauma that needs to have the central nasal complex repaired and has been nasally intubated, the conversion of the NTT to an OTT without extubating has been proposed to avoid the risks associated with removing a secure airway intraoperatively [13]. 

In short, standard nasal intubation is performed, and the intra-oral procedure is completed. The nasal tube is then cut external to the nares, distal to the pilot tube, and delivered orally. Mittal et al. [23] performed the nasal to oral switch in 161 patients for the purpose of nasal bone reduction after the fixation of the maxilla and mandible. Fiberoptic guidance for nasal intubation was required in 52 patients either because of cervical spine injury or a difficult airway. Nasal bleed was the most common complication reported in 20 cases. No cases of meningitis or the intracranial passage of the tube were reported.

There are several advantages of this technique over current options. In a patient who does not otherwise require tracheostomy, both reintubation and a delayed procedure with a second general anesthetic may be avoided. Nasal fractures can also then be treated in the same surgical procedure without a nasal tube in place.

The contraindications for this tube switch procedure may include severe frontobasilar fractures, severe midface fractures, the inability to achieve the rigid fixation of facial bone segments necessitating IMF, and planned prolonged postoperative intubation. Gross distortion/wounds in the nasopharynx or oropharynx may also preclude a safe switch.

### 4.4. Is Submental Intubation the First Choice in Panfacial Fractures?

Submental intubation was first described by Hernández-Altemir in 1984 [29] and reported in the English literature in 1986 [30]. Since its development, it has been used as a first-choice technique for intraoperative airway management in complex maxillofacial injuries by some, with panfacial trauma being one of the most common indications for submental endotracheal intubation [31,32,33,34,35,36,37,38,39,40,41,42,43,44,45,46].

An OTI is performed, and then the tube is passed through the anterior floor of the mouth and reconnected to the ventilator. This allows access to the lower two-thirds of the face without interference.

In a systematic review performed by Goh et al. [47] that included 2229 patients, the indication for this technique was maxillofacial trauma in 81% of cases. The mean intubation time was 10 min. The complication rate was 7%, with superficial skin infection being the most reported complication.

The most significant complication after SMI is accidental extubation [31] or the accidental perforation of the pilot balloon in 4.35% of cases [42]. Other complications include wound infections, 2–3.5% [37,41]; transient lingual and submental nerve paresthesia, bleeding, and submental hypertrophic scar, 1.45–3.57% [32,35]; orocutaneous fistula; traumatic injuries to the submandibular and sublingual glands or ducts [36]; and mucocele formation.

Submental intubation appears to have lower morbidity and better outcomes when compared with tracheostomy. In a prospective, randomized, controlled trial of 32 patients with panfacial fractures by Emara et al. [9], randomly assigned to elective tracheostomy or SMI, the average time required to perform SMI was 8.35 min versus 30.75 min to perform an elective surgical tracheostomy. No complications were reported with SMI, whilst in the elective tracheostomy group, surgical emphysema was reported in two patients. The submental scar was acceptable in all patients, while the tracheostomy scar needed revision in four cases.

Contraindications for SMI are the need for long-term ventilatory support and maintenance and the need for multiple maxillofacial surgical procedures. If mechanical ventilation or intubation is required postoperatively, SMI could be switched over back to a standard OTT, but it has been maintained in a postoperative setting for up to 2 days with good tolerance [43].

In the presence of a comminuted symphyseal or parasymphyseal fracture in which an external approach is needed, SMI should be avoided, as the tube may interfere with the surgical approach or reduction. Gadre and Waknis [14] noted that in patients with comminuted fractures in the symphysis and parasymphyseal regions, the conventional submental technique will necessitate the stripping of the lingual periosteum which would be detrimental to the blood supply of smaller fragments, and they proposed using the area between the two mandibular molars on the contralateral side to the fracture for access [48].

The presence of cervical hematoma, infection, and severe swelling in the anterior neck are relative contraindications to SMI.

In summary, SMI is a minimally invasive procedure that is easier to perform and can be completed rapidly with a success rate reported to be 100% and with a complication rate that ranges from 0 to 7% [47].

### 4.5. Is There Still a Role for Tracheostomy for Airway Management in Craniomaxillofacial Trauma?

A tracheostomy is a definitive airway that does not interfere with surgical access to the face, by virtue of being placed more caudally. It is a traditional approach to airway management in complex reconstructive procedures that is usually considered to be safe [15]. It was the first choice for patients with panfacial fractures or those with loss of consciousness and midface fractures in a survey of surgeons and anesthesiologists [7].

Holmgren et al. [49] reported that 11.6% of all facial fracture patients received a tracheostomy during the same operative procedure. The patients who had a tracheostomy performed had a lower Glasgow Coma Scale score, and the pattern of fractures were different from those that did not have a tracheostomy, with a significantly higher incidence of mandible, multiple mandible, Le Fort III, and laryngeal fractures. There were no known cases of glottic or subglottic stenosis, severe bleeding requiring a return to the operating room, airway obstruction, or the loss of a secured airway.

Because of the improved airway management techniques available, the use of rigid internal fixation may obviate the need for IMF or at least reduce the period that it is needed, and many surgeons no longer advocate for routine tracheostomy for patients with complex facial trauma [8,9,10,15,22,23]. Tracheostomy is indicated in cases with prolonged ventilation and where SMI, OTI, and NTI are contraindicated or in patients admitted with pre-existing cricothyroidotomy. Head and neck trauma is the most common injury requiring prolonged mechanical ventilation, and in patients with head and neck trauma, ventilator-associated pneumonia was reported as the major cause of death [50].

A tracheostomy is also indicated in polytrauma where a patient might require several operations, on the face or other body areas, over a relatively short period of time and obviates the need for repeated tracheal intubation.

Although a tracheostomy is a secure airway, it is associated with a significant number of intraoperative and postoperative complications [51]. These complications can be grouped into intraoperative, early (<1 week), and late complications. Reported intraoperative complications are nerve injury, bleeding, and the development of subcutaneous emphysema or a pneumomediastinum, following the passage of the tracheostomy tube into a false lumen. Tube blockage, respiratory infection, aspiration, and pneumonia are reported during the early postoperative period. Tracheal stenosis, tracheomalacia, tracheoesophageal fistula, voice changes, tracheal granulomas, and unfavorable scar are the late complications reported.

Intraoperative, early (<1 week), and late complication rates were reported to be 1.4%, 5.6%, and 7.1%, respectively. Postoperative bleeding was identified as the most common early complication (2.6%), whereas airway stenosis was the most common late complication (1.7%).

In a series of 1138 tracheostomies, Goldenberg et al. [52] reported 49 major complications (4.3%), including 4 cases of accidental decannulation, 2 cases of trachea-innominate artery fistula with subsequent fatal hemorrhage, and 2 cases of postoperative tension pneumothorax with a mortality of 0.7% (8 patients) directly related to the tracheotomy.

An alternative that can be considered to open tracheostomy is percutaneous tracheostomy [53]. This technique has gained widespread popularity, often replacing conventional surgical tracheostomy in intensive care units. In highly trained teams, percutaneous tracheostomy can be performed in 5 min, which compares favorably to open tracheostomy. Fiberoptic assistance is recommended.

Absolute contraindications to percutaneous tracheostomy include cervical instability, uncontrolled coagulopathy, and infection at the planned insertion site or tracheomalacia. Relative contraindications include difficult anatomy (short neck, overlying blood vessels, morbid obesity, minimal neck extension, or tracheal deviation).

Life-threatening complications, including major bleeding, or problems with the tracheostomy tube [54] may also arise with percutaneous tracheostomy, but it results in fewer wound infections and better esthetic scars when compared to open tracheostomy [55].

It is possible to use a percutaneous tracheostomy for postoperative airway suctioning and it may be used for extended intubation after elective head and neck surgery [56]. There is only one report of its use in a postoperative setting of patients with panfacial trauma whose intermaxillary fixation was not to be removed [57].

### 4.6. What Is the Role of FONA with Elective Cricothyroidotomy?

Surgical cricothyroidotomy is a surgical airway technique in which an airway device is inserted into the trachea through an incision made in the cricothyroid membrane.

Surgical cricothyroidotomy is traditionally an emergency procedure and is recommended as the safest emergency surgical airway technique by the Difficult Airway Society [4]. Its role as an elective surgical airway in maxillofacial trauma is seldom reported [58]. A cricothyroidotomy, when present, is normally converted to a tracheostomy.

One of the major complications attributed to this technique is subglottic stenosis. An incidence of 0.5% of subglottic stenosis after surgical cricothyroidotomy was reported by Teo et al. [58], which compares favorably with the incidence of serious complications of tracheostomy.

Kuroiwa et al. [16] reported three cases of elective surgical cricothyroidotomy for anesthetic management during the surgical repair of maxillofacial injuries involving a basal skull fracture or nasal bone fracture. No major complications, such as subglottic stenosis or voice change, occurred.

### 4.7. Staged Treatment of Panfacial Fractures

Although not a technique of airway management per se, the staging treatment of the different thirds of the face in a panfacial fracture can bypass some of the difficulties the airway poses to the management of these fractures.

This usually consists of restoring the lower third in the first operation and addressing the middle and upper thirds in the second operation. This can benefit the patient by requiring a shorter time in the operating room and gives the surgeon more time for the planning of the upper part of the face. Moreover, a stable mandible makes the planning of the midface much easier. The first operation may be performed with a standard NTT, which is removed at the end of this procedure and can either be replaced with an alternative such as OTI, if the occlusion does not need assessing, or SMI for the treatment of the upper and middle thirds.

### 4.8. Algorithm Proposal for Management of Airway in Panfacial Fractures (Figure 1)

The first consideration will be whether there is already an airway device in situ from the acute or ongoing management of the patient. When a tracheostomy, either open or percutaneous, is already in situ, this can normally be utilized. If the patient arrives at the operating room with a cricothyroidotomy, its use can be assessed depending on the type of surgery and the anesthetic evaluation of the case. If it is not deemed adequate, then it is converted to a surgical tracheostomy. When an endotracheal tube is in situ, the decision to change this can be based on the proposed algorithm, after due consideration of the risks associated with tube changes, if one is indicated.

The second consideration is whether the occlusion needs to be assessed or whether IMF might be required intraoperatively or immediately postoperatively. When the occlusion must be assessed, it is still possible to proceed with an OTT in place, by moving this to a retromolar position, but this will depend on a suitable OTT being in situ, there being sufficient space available in the retromolar area, and also on the absence of interference in the reduction of the fractures. The preferred method when the occlusion needs to be established is NTI. Although there are concerns about using an NTT when there are significant skull base fractures, there does not appear to be a significant risk in this technique. When possible, fiberoptic-guided NTI should be used. Submental intubation is the best option when any contraindications to NTI exist or when a severe nasoethmoid fracture makes NTI impossible.

The final consideration is the need for either prolonged intubation or repeated anesthetics over a short period, in which case FONA will be the most common choice, unless there is a specific contraindication.

When it is expected that a patient will be extubated immediately postoperatively, or shortly afterwards, and should not require multiple procedures, an endotracheal tube is the simplest and safest option. The factors that influence the choice of airway in this setting include the need to establish the occlusion and the involvement of the central nasal complex. In the unusual circumstance that the occlusion does not need to be established intraoperatively, a simple OTT will provide an easy and safe secure airway.

**Figure 1:**
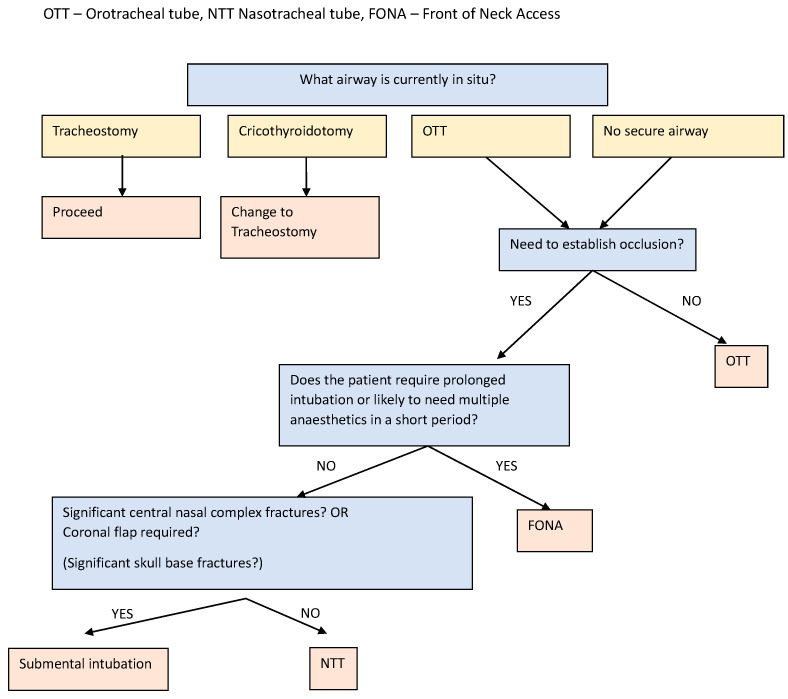
Algorithm for airway management in panfacial fractures.

## 5. Conclusions

Obtaining and securing a definitive airway in patients with panfacial fractures can be challenging. An airway may be required during the acute management phase, where options may be limited by the availability of skill sets and equipment. In patients with only maxillofacial injuries, the details of the injury can guide the choice of airway, but panfacial fractures are commonly associated with intra-cerebral and cervical spine injuries and polytrauma, necessitating either prolonged intubation or multiple anesthetics over a relatively short period of time.

When an occlusion needs to be assessed or intermaxillary fixation required, the airway device cannot pass through the mouth, unless it is placed retromolar. This is, however, not routinely used because of difficulties placing the device without interfering with surgical access or the reduction of fractures.

To avoid interference with the occlusion, NTI has become the norm in the management of facial fractures. There continues to be concern about the use of NTI in skull base fractures although it has been shown to be safe when performed under fiberoptic guidance. The main problem is that the tube then passes over the top of the head and interferes with coronal access, and its position makes the reduction of nasal complex fractures more difficult.

Changing from NTI to an OTT intraoperatively can circumvent these problems. This can be conducted by completely removing the NTT and immediately re-inserting an OTT but carries the risks associated with an anaesthetized patient not having a secure airway during the change. This risk can be avoided by keeping the NTT and repositioning it trans-orally, but this technique can be troublesome when there are significant soft tissue or mandibular/dentoalveolar injuries.

Submental intubation is increasingly reported in panfacial fractures as it is relatively safe and easy to perform and enables free access to all areas of the facial skeleton unless extra-oral access to an anterior mandible fracture is required. Its main contraindication is the need for prolonged intubation postoperatively.

Surgical tracheostomy remains the method of choice for securing the airway in patients in need of prolonged intubation or multiple anesthetics over a relatively short period of time. Percutaneous tracheostomy can be used instead of open tracheostomy and has a slightly improved complication rate, with a few additional contraindications. Cricothyroidotomy can be an alternative to tracheostomy and has a low complication rate, but it is not normally considered for definitive airway management in trauma patients.

Staged surgery can avoid some of these controversies, but its role is more related to the appropriate planning and timing of different surgical procedures.

## References

[1] Massenburg B.B., Lang M.S. (2021). Management of Panfacial Trauma: Sequencing and Pitfalls. Semin. Plast. Surg..

[2] Wenig B.L. (1991). Management of panfacial fractures. Otolaryngol. Clin. N. Am..

[3] Perry M., Morris C. (2008). Advanced trauma life support (ATLS) and facial trauma: Can one size fit all? Part 2: ATLS, maxillofacial injuries and airway management dilemmas. Int. J. Oral Maxillofac. Surg..

[4] Lockey D., Crewdson K., Weaver A., Davies G. (2014). Observational study of the success rates of intubation and failed intubation airway rescue techniques in 7256 attempted intubations of trauma patients by pre-hospital physicians. Br. J. Anaesth..

[5] Frerk C., Mitchell V.S., McNarry A.F., Mendonca C., Bhagrath R., Patel A., O’Sullivan E.P., Woodall N.M., Ahmad I. (2015). Difficult Airway Society intubation guidelines working group. Difficult Airway Society 2015 guidelines for management of unanticipated difficult intubation in adults. Br. J. Anaesth..

[6] Wang L., Lee T.S., Wang W., Yi D.I., Sokoya M., Ducic Y. (2019). Surgical Management of Panfacial Fractures. Facial Plast. Surg..

[7] Smoot E.C., Jernigan J.R., Kinsley E., Rey R.M. (1997). A survey of operative airway management practices for midface fractures. J. Craniofac. Surg..

[8] Kolte V.S., Shenoi R.S., Ingole P.D., Karmarkar J.S., Rajguru J.G., Deole S.S. (2021). Finding a way for airway: A retrospective study. Minerva Surg..

[9] Emara T.A., El-Anwar M.W., Omara T.A., Anany A., Elawa I.A., Rabea M.M. (2019). Submental intubation versus tracheostomy in maxillofacial fractures. Oral Maxillofac. Surg..

[10] Daniels J.S., Albakry I., Braimah R.O., Samara M.I., Albalasi R.A., Begum F., Al-Kalib M.A. (2020). Experience with Airway Management and Sequencing of Repair of Panfacial Fractures: A Single Tertiary Healthcare Appraisal in Najran, Kingdom of Saudi Arabia—A Retrospective Study. Ann. Maxillofac. Surg..

[11] Sittitavornwong S., Mostofi P., DiLuzio K., Kukreja P., Deatherage H., Kukreja P. (2021). Does the Retromolar Area Provide Adequate Space for an Oral Endotracheal Tube Without Interfering With Intermaxillary Fixation?. J. Oral Maxillofac. Surg..

[12] Marlow T.J., Goltra D.D., Schabel S.I. (1997). Intracranial placement of a nasotracheal tube after facial fracture: A rare complication. J. Emerg. Med..

[13] Werter J.R., Richardson G., Mcilwain M.R. (1994). Nasal tube switch: Converting from nasal to an oral endotracheal tube without extubation. J. Oral Maxillofac. Surg..

[14] Gadre K.S., Waknis P.P. (2010). Transmylohyoid/submental intubation: Review, analysis, and refinements. J. Craniofac Surg..

[15] Taicher S., Givol N., Peleg M., Ardekian L. (1996). Changing indications for tracheostomy in maxillofacial trauma. J. Oral Maxillofac. Surg..

[16] Kuroiwa M., Kumazawa K., Ito S., Arai M., Okamoto H. (2015). Elective use of surgical cricothyroidotomy for maxillofacial fracture fixation with contraindication of nasotracheal intubation: A case report. JA Clin. Rep..

[17] Vidya B., Cariappa K.M., Kamath A.T. (2012). Current perspectives in intra operative airway management in maxillofacial trauma. J. Maxillofac. Oral Surg..

[18] Bonfils P. (1983). Difficult intubation in Pierre-Robin children, a new method: The retromolar route. Der Anaesthesist..

[19] Martinez-Lage J.L., Eslava J.M., Cebrecos A.I., Marcos O. (1998). Retromolar intubation. J. Oral Maxillofac. Surg..

[20] Truong A.T., Truong D.T. (2015). Retromolar flexible fiber-optic orotracheal intubation: A novel alternative to nasal intubation and tracheostomy in severe trismus. Head Neck.

[21] Abramson S.I., Holmes A.A., Hagberg C.A. (2008). Awake Insertion of the Bonils Retromolar Intubation Fiberscope^TM^ in Five Patients with Anticipated Dificult Airways. Anesth. Analg..

[22] Raval C.B., Rashiduddin M. (2011). Airway management in patients with maxillofacial trauma—A retrospective study of 177 cases. Saudi J. Anaesth..

[23] Mittal G., Mittal R.K., Katyal S., Uppal S., Mittal V. (2014). Airway management in maxillofacial trauma: Do we really need tracheostomy/submental intubation. J. Clin. Diagn. Res..

[24] Bahr W., Stoll P. (1992). Nasal intubation in the presence of frontobasal fractures: A retrospective study. J. Oral Maxillofac. Surg..

[25] Goodisson D.W., Shaw G.M., Snape L. (2001). Intracranial intubation in patients with maxillofacial injuries associated with base of skull fractures?. J. Trauma.

[26] Rhee K.J., Muntz C.B., Donald P.J., Yamada J.M. (1993). Does nasotracheal intubation increase complications in patients with skull base fractures?. Ann. Emerg. Med..

[27] Jazayeri-Moghaddas O.P., Tse W., Herzing K.A., Markert R.J., Gans A.J., McCarthy M.C. (2017). Is nasotracheal intubation safe in facial trauma patients?. Am. J. Surg..

[28] Rosen C.L., Wolfe R.E., Chew S.E., Branney S.W., Roe E.J. (1997). Blind nasotracheal intubation in the presence of facial trauma. J. Emerg. Med..

[29] Hernández-Altemir F. (1984). Una nueva técnica de intubación endotraqueal (vía submental). Rev. Iberoamer. Cirug. Oral Maxilof..

[30] Hernández Altemir F. (1986). The submental route for endotracheal intubation. A new technique. J. Maxillofac. Surg..

[31] Bhola N., Jadhav A., Kala A., Deshmukh R., Bhutekar U., Prasad G.S.V. (2017). Anterior Submandibular Approach for Transmylohyoid Endotracheal Intubation: A Reappraisal with Prospective Study in 206 Cases of Craniomaxillofacial Fractures. Craniomaxillofac Trauma Reconstr..

[32] Caron G., Paquin R., Lessard M.R., Trépanier C.A., Landry P.E. (2000). Submental endotracheal intubation: An alternative to tracheotomy in patients with midfacial and panfacial fractures. J. Trauma.

[33] Kaiser A., Semanoff A., Christensen L., Sadoff R., DiGiacomo J.C. (2018). Submental Intubation: An Underutilized Technique for Airway Management in Patients with Panfacial Trauma. J. Craniofac. Surg..

[34] Rodrigues W.C., de Melo W.M., de Almeida R.S., Pardo-Kaba S.C., Sonoda C.K., Shinohara E.H. (2017). Submental Intubation in Cases of Panfacial Fractures: A Retrospective Study. Anesth. Prog..

[35] Banerjee P.K., Jain A., Behera B. (2016). Submandibular intubation as an alternative for intra-operative airway management in maxillofacial fractures—Our institutional experience. Indian J. Anaesth..

[36] Kumar K.A., Kumar B.P., Mohan A.P., Masram A.K., Tyro D., Gandla D. (2015). Assessment of the Efficacy of Submental Intubation in the Management of Midfacial and Panfacial Trauma Patients. J. Maxillofac. Oral Surg..

[37] Prakash V.J., Chakravarthy C., Attar A.H. (2014). Submental/Transmylohyoid route for endotracheal intubation in maxillofacial surgical procedures: A review. J. Int. Oral Health.

[38] Shenoi R.S., Badjate S.J., Budhraja N.J. (2011). Submental orotracheal intubation: Our experience and review. Ann. Maxillofac. Surg..

[39] Shetty P.M., Yadav S.K., Upadya M. (2011). Submental intubation in patients with panfacial fractures: A prospective study. Indian. J. Anaesth..

[40] Tidke A.S., Borle R.M., Madan R.S., Bhola N.D., Jadhav A.A., Bhoyar A.G. (2013). Transmylohoid/Submental Endotracheal Intubation in Pan-facial Trauma: A Paradigm Shift in Airway Management with Prospective Study of 35 Cases. Indian J. Otolaryngol. Head Neck Surg..

[41] Szantyr A., Szuta M., Zapała J. (2016). Airway management using submental intubation in head and neck surgery. Folia Med. Cracov..

[42] Mishra R., Yadav D., Tripathi S., Kandel L., Baral P.P., Shubham S., Karn A., Dutta K. (2020). Submental Intubations in Panfacial Fractures. Clin. Cosmet. Investig. Dent..

[43] Anwer H.M., Zeitoun I.M., Shehata E.A. (2007). Submandibular approach for tracheal intubation in patients with panfacial fractures. Br. J. Anaesth..

[44] Singaram M., Ganesan I., Kannan R., Kumar R. (2016). Evaluation of safety and usefulness of submental intubation in panfacial trauma surgery. J. Korean Assoc. Oral Maxillofac. Surg..

[45] Garg M., Rastogi B., Jain M., Chauhan H., Bansal V. (2010). Submental intubation in panfacial injuries: Our experience. Dent. Traumatol..

[46] Taglialatela Scafati C., Maio G., Aliberti F., Taglialatela Scafati S., Grimaldi P.L. (2006). Submento-submandibular intubation: Is the subperiosteal passage essential ? Experience in 107 consecutive cases. Br. J. Oral Maxillofac. Surg..

[47] Goh E.Z., Loh N.H.W., Loh J.S.P. (2020). Submental intubation in oral and maxillofacial surgery: A systematic review 1986–2018. Br. J. Oral Maxillofac. Surg..

[48] Gadre K.S., Kushte D. (1992). Transmylohyoid oroendotracheal intubation: A novel method. J. Craniofac. Surg..

[49] Holmgren E.P., Bagheri S., Bell R.B., Bobek S., Dierks E.J. (2007). Utilization of tracheostomy in craniomaxillofacial trauma at a level-1 trauma center. J. Oral Maxillofac. Surg..

[50] Kung S.C., Lin W.T., Tsai T.C., Lin M.H., Chang C.H., Lai C.C., Chao C.M. (2017). Epidemiologic characteristics, and outcomes of major trauma patients requiring prolonged mechanical ventilation. Medicine.

[51] Halum S.L., Ting J.Y., Plowman E.K., Belafsky P.C., Barbarger C.F., Postma G.N., Pitman M.J., LaMonica D., Moscatello A., Khosla S. (2012). A multi-institutional analysis of tracheotomy complications. Laryngoscope.

[52] Goldenberg D., Ari E.G., Golz A., Danino J., Netzer A., Joachims H.Z. (2000). Tracheotomy complications: A retrospective study of 1130 cases. Otolaryngol. Head Neck Surg..

[53] Susarla S.M., Peacock Z.S., Alam H.B. (2012). Percutaneous dilatational tracheostomy: Review of technique and evidence for its use. J. Oral Maxillofac. Surg..

[54] Brass P., Hellmich M., Ladra A., Ladra J., Wrzosek A. (2016). Percutaneous techniques versus surgical techniques for tracheostomy. Cochrane Database Syst. Rev..

[55] Ikegami Y., Iseki K., Nemoto C., Tsukada Y., Shimada J., Tase C. (2014). Patient questionnaire following closure of tracheotomy fistula: Percutaneous vs. surgical approaches. J. Intensive Care.

[56] Gaukroger M.C., Allt-Graham J. (1994). Percutaneous dilatational tracheostomy. Br. J. Oral Maxillofac. Surg..

[57] Arabi Y., Haddad S., Al Tuwairgi O. (2006). Percutaneous tracheostomy in a patient with mandibulo-maxillary interfixation with modified approach for bronchoscopic guidance—Case report. Middle East. J. Anaesthesiol..

[58] Teo N., Garrahy A. (2013). Elective surgical cricothyroidotomy in oral and maxillofacial surgery. Br. J. Oral Maxillofac. Surg..

